# The hierarchy quorum sensing network in *Pseudomonas aeruginosa*

**DOI:** 10.1007/s13238-014-0100-x

**Published:** 2014-09-25

**Authors:** Jasmine Lee, Lianhui Zhang

**Affiliations:** 1 Institute of Molecular and Cell Biology, Agency for Science, Technology and Research, Singapore, Singapore; 2 Guangdong Province Key Laboratory of Microbial Signals and Disease Control, College of Natural Resources and Environmental Sciences, South China Agricultural University, Guangzhou, 510642 China

**Keywords:** quorum sensing, IQS, PQS, las, rhl, *Pseudomonas aeruginosa*, virulence, environmental factors

## Abstract

*Pseudomonas aeruginosa* causes severe and persistent infections in immune compromised individuals and cystic fibrosis sufferers. The infection is hard to eradicate as *P. aeruginosa* has developed strong resistance to most conventional antibiotics. The problem is further compounded by the ability of the pathogen to form biofilm matrix, which provides bacterial cells a protected environment withstanding various stresses including antibiotics. Quorum sensing (QS), a cell density-based intercellular communication system, which plays a key role in regulation of the bacterial virulence and biofilm formation, could be a promising target for developing new strategies against *P. aeruginosa* infection. The QS network of *P. aeruginosa* is organized in a multi-layered hierarchy consisting of at least four interconnected signaling mechanisms. Evidence is accumulating that the QS regulatory network not only responds to bacterial population changes but also could react to environmental stress cues. This plasticity should be taken into consideration during exploration and development of anti-QS therapeutics.

## Introduction

*Pseudomonas aeruginosa* is a ubiquitous, gram-negative bacterium that thrives in diverse habitats and environments. Usually a commensal on the host body, *P. aeruginosa* is capable of transforming into an opportunistic pathogen when there is a breach of host tissue barriers or a suppressed immune system (Van Delden and Iglewski, 1998). *P. aeruginosa* is an important nosocomial pathogen, affecting a wide category of patients convalescing in hospitals. They include patients with cystic fibrosis and other lung diseases, traumatized cornea, burns, Gustilo open fractures, long-term intubated patients, the immune-compromised and elderly individuals. The infections caused by *P. aeruginosa* are usually resistant to treatment by multiple antibiotics and can lead to severe and persistent infections (Bonomo and Szabo, 2006; Chernish and Aaron, 2003; Doshi et al., 2011; Tan, 2008). This translates into further complications and secondary fungal infections, extension of hospital stay, therapeutic failure, and in some cases, premature death of cystic fibrosis patients (Henry et al., 1992; Kosorok et al., 2001; Rabin et al., 2004; Tan, 2008). Because *P. aeruginosa* grows and survives in various environmental conditions, it makes acquiring an infection extremely easy and outbreaks of extreme drug-resistant strains are common among hospital wards and intensive care units.

**Figure 1:**
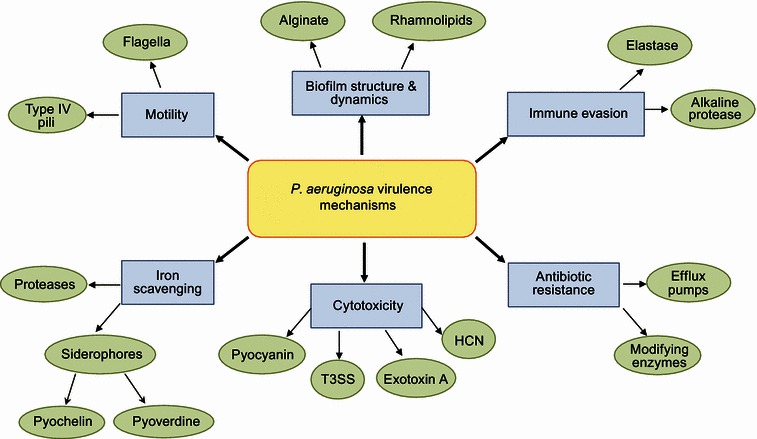
Virulence mechanisms employed during *P. aeruginosa* infections

It is believed that understanding the regulatory mechanisms with which *P. aeruginosa* governs virulence gene expression may hold the key to develop alternative therapeutic interventions to control and prevent the bacterial infections (Fig. 1). The recent research progresses show that a bacterial cell-cell communication mechanism, widely known as quorum sensing (QS), plays a key role in modulating the expression of virulence genes in *P. aeruginosa*. The term quorum sensing was proposed two-decades ago by three renowned microbiologists based on the bacterial population density-dependent regulatory mechanisms found in several microbial organisms, including *Vibrio fischeri*, *Agrobacterium tumefaciens*, *P. aeruginosa* and *Erwinia carotovora* (Fuqua et al., 1994). Since then, various QS systems have been found in many bacterial pathogens, which are commonly associated with the regulation of virulence gene expression and biofilm formation (Deng et al., 2011; Ng and Bassler, 2009; Pereira et al., 2013; Whitehead et al., 2001). Typically, quorum sensing bacteria produce and release small chemical signals, and at a high population density, the accumulated signals interact with cognate receptors to induce the transcriptional expression of various target genes including those encoding production of virulence factors. While QS becomes a popular concept, it is worthy to note that opinions arose on whether QS is the most-fitted term for mechanistic explanation of the above-mentioned bacterial group behavior. The point of contention stemmed from the fact that autoinducer concentration, the key determinant of “quorum” as defined by QS, was not simply a function of bacterial cell density, but a combined output of many factors such as diffusion rate and spatial distribution, and hence alternative terms such as “diffusion sensing”, “efficiency sensing” and “combinatorial quorum sensing” were proposed (Hense et al., 2007; Redfield, 2002; Cornforth et al., 2014). Whilst interesting, these alternative opinions await further experimental endorsement and by far QS remains as the most rigorously tested mechanism of bacteria cell-cell communication and collective responses.

Given its importance as a human pathogen, *P. aeruginosa* has been the subject of intensive investigations and become one of the model organisms in QS research. The research progresses in the last two decades have unveiled a sophisticated hierarchy QS network in this pathogen, which consists of a few sets of connected systems, including *las*, *iqs*, *pqs* and *rhl*. Particularly, recent findings show that the QS network in *P. aeruginosa* is highly adaptable and capable of responding to external biostress cues, which provides the pathogen flexibility in the control of virulence gene expression. It would not be surprising that other bacterial pathogens may have also evolved similar flexible QS systems which could respond to changed environmental conditions. This is an important factor to consider in the development of quorum sensing inhibitors (QSIs) as therapeutics, since bacteria routinely encounters adverse environmental conditions when infecting host organisms. This review will provide an overview on the QS systems in *P. aeruginosa*, focusing on a recently discovered integrated quorum sensing system (IQS), and on the interactions between all the four QS systems and how environmental cues could affect the QS hierarchy.

## Quorum Sensing Systems in *Pseudomonas aeruginosa*

### History of quorum sensing

The concept of quorum sensing in *P. aeruginosa* was an extension of the studies based on the prototype *luxI*-*luxR* system in *Vibrio fischeri*, in which *luxI* encodes the biosynthesis of an acylhomoserine lactone (AHL) signal *N*-(3-oxohexanoyl)-L-homoserine lactone (OHHL), and *luxR* encodes an AHL-dependent transcription factor (Eberhard, 1972; Nealson et al., 1970; Stewart and Williams, 1992; Williams et al., 1992). With significant homology to the LuxR protein, LasR in *P. aeruginosa* was initially identified to be a key regulator in the expression of *lasB* gene encoding for a metalloprotease elastase (Cook, 1992; Gambello and Iglewski, 1991). Subsequently, LasR was also shown to be required for the transcription of *aprA*, *lasA* and *toxA*, and thus it was thought to be a global regulator of the virulence genes in *P. aeruginosa* (Gambello and Iglewski, 1991; Gambello et al., 1993; Passador et al., 1993; Toder et al., 1991). LasI, the LuxI equivalent in *P. aeruginosa*, was proposed to synthesize AHL signals with autoinducing and elastase-regulating properties (Jones et al., 1993). One year later, the actual chemical structure of this *Pseudomonas* autoinducer (PAI) was characterized as *N*-(3-oxododecanoyl)-homoserine lactone (OdDHL) (Pearson et al., 1994). PAI is structurally related to the autoinducers discovered in other gram-negative bacteria species (Cao and Meighen, 1993; Eberhard et al., 1981; Zhang et al., 1993).

Shortly after, a second autoinducer, factor 2, was discovered in *P. aeruginosa* (Pearson et al., 1995). This discovery was made following a puzzling observation that an unusually high concentration of OdDHL was required to activate the *lasB* promoter (Pearson et al., 1995), suggesting that another factor in PAO1 may be required for *lasB* activation. The *P. aeruginosa* factor 2 was structurally identified to be *N*-butyrylhomoserine lactone (BHL) (Pearson et al., 1995). BHL was not shown to interact with LasR protein directly to activate *lasB* gene expression, nor does it directly regulate the latter (Pearson et al., 1995), triggering another hunt for its cognate receptor. Within the same year, RhlR, a regulatory protein encoded by the rhamnolipid synthase gene cluster *rhlABR*, was identified to be the cognate receptor of BHL (Ochsner and Reiser, 1995). The *rhlI* gene, which encodes the biosynthesis of BHL and sharing significant sequence homologies to *luxI* and *lasI*, was found at the downstream of the *rhlABR* cluster. Expression of RhlI could restore the production of several exoproducts such as elastase, pyocyanin, hemolysin and rhamnolipids, and both RhlI and RhlR are required for the full activation of the *rhlABR* and *lasB* promoters (Brint and Ohman, 1995; Ochsner and Reiser, 1995).

### The *las* and *rhl* quorum sensing systems

These key discoveries in *P. aeruginosa* QS systems inspired further researches on their functions, regulons and the molecular mechanisms with which the *las* and *rhl* circuits activate the expression of QS-responsive genes. The results showed that upon binding with the respective autoinducers OdDHL and BHL, the receptor proteins LasR and RhlR get activated and form complexes. The LasR-OdDHL and RhlR-BHL complexes bind to the conserved *las-rhl* boxes residing in the promoters of target genes, thereby activating their transcriptional expression (Schuster and Greenberg, 2007; Whiteley and Greenberg, 2001; Whiteley et al., 1999). Transcriptomic studies based on *lasI* and *rhlI* mutants revealed that the regulons are on a continuum, with some genes that respond dramatically well to OdDHL (e.g. *lasA*), some with BHL specificities (e.g. *rhlAB*), and some equally well to both signals (Schuster and Greenberg, 2006; Schuster et al., 2003). These genes constitute nearly 10% of *P. aeruginosa* genome, and therefore accounts for a majority of the physiological processes and virulence phenotypes (Schuster and Greenberg, 2006). Some of these key virulence genes are listed for the convenience of discussion (Table 1).

**Table 1:** Examples of quorum sensing (QS) regulated virulence factors and their effects to the human host

QS regulated gene	Protein or virulence factor	Effects to host during infections	Benefits to *P. aeruginosa*	References
*lasB*	Elastase	Degradation of elastin, collagen, and other matrix proteins	Extracellular iron acquisition from host proteins	Wolz et al. (1994); Yanagihara et al. (2003)
*lasA*	Protease	Disruption of epithelial barrier	Staphylolytic activity, host immune evasion and enhanced colonization	Kessler et al. (1993); Park et al. (2000)
*toxA*	Exotoxin A	Cell death	Establishment of infection; enhanced colonization	Daddaoua et al. (2012); McEwan et al. (2012)
*aprA*	Alkaline protease	Degradation of host complement system and cytokines	Immune evasion and persistent colonization	Laarman et al. (2012)
*rhlAB*	Rhamnosyl-transferases (rhamnolipids)	Necrosis of host macrophage and polymorphonuclear lymphocytes	Immune evasion; biofilm development	Jensen et al. (2006); Lequette and Greenberg (2005)
*lecA*	Lectin (galactophilic lectin)	Paralysis of airway cilia	Establishment of infection; enhanced colonization	Adam et al. (1997)
*hcnABC*	Hydrogen cyanide	Cellular respiration arrest; Poorer lung function	Enhanced colonization	Ryall et al. (2008); Solomonson (1981)
*phzABCDEFG*, *phzM*	Pyocyanin	Oxidative effects dampen host cellular respiration and causes oxidative stress; Paralysis of airway cilia; Delayed inflammatory response to *P. aeruginosa* infections through neutrophil damage	Establishment of infection; enhanced colonization; immune evasion	Denning et al. (1998); Jackowski et al. (1991); Lau et al. (2004)

LasR also induces the expression of RsaL, a transcriptional repressor of *lasI*. Binding of RsaL to the bidirectional *rsaL-lasI* promoter inhibits the expression of both genes, which generates a negative feedback loop that counteracts the positive signal feedback loop mentioned earlier, thereby balancing the levels of OdDHL (Rampioni et al., 2007). Whilst LasR/OdDHL and RsaL do not compete for the same binding site on the *lasI* promoter region, the repression by RsaL is stronger than the activation by LasR (Rampioni et al., 2007). RsaL also inhibits the expression of some QS target genes such as biosynthetic genes of pyocyanin and cyanide (Rampioni et al., 2007). A range of positive and negative regulatory proteins were subsequently identified and they control the *las* and *rhl* systems in a variety of ways. Noteworthy are the regulatory effects of QscR and VqsR, which are homologues of LuxR. QscR forms heterodimers with LasR/OdDHL and RhlR/BHL and prevents their binding with the promoter DNA of downstream responsive genes, therein dampening the *las* and *rhl* QS signalling effects (Ledgham et al., 2003a). QscR also binds to OdDHL and utilize it for activating its own regulon (Chugani et al., 2001; Fuqua, 2006; Schuster and Greenberg, 2006). VqsR is a positive regulator of the *las* QS system and is itself regulated by the LasR/OdDHL complex (Li et al., 2007). More recently, an anti-activator QslA was identified, which binds to LasR via protein-protein interaction and prevents the interaction of the latter with promoter DNA of the *las* responsive genes. The inhibitory effect of QslA on LasR is irrespective of OdDHL concentrations. By disrupting the ability of LasR to trigger the expression of downstream genes and cause a QS response, QslA controls the overall QS activation threshold (Seet and Zhang, 2011). There are quite a few other super-regulators of the AHL-based QS systems which are summarized in the table below (Table 2). In addition, quorum quenching enzymes, which degrade AHL signals, the AHL-acylases PvdQ and QuiP, are also involved in balancing the level of AHL signals in *P. aeruginosa* (Huang et al., 2006; Sio et al., 2006).

**Table 2:** Super-regulators of QS in *P. aeruginosa*

Regulator	Mechanism of action	References
AlgR2	Negative transcriptional regulator of *lasR* and *rhlR*	Ledgham et al. (2003a); Westblade et al. (2004)
DksA	Negative transcriptional regulator of *rhlI*	Branny et al. (2001); Jude et al. (2003); van Delden et al. (2001)
GacA/GacS	Positive transcriptional regulator of *lasR* and *rhlR*	Parkins et al. (2001); Reimmann et al. (1997)
MvaT	Negative transcriptional regulator (global regulation)	Diggle et al. (2002)
QscR	Negative regulator (anti-activator) of LasR protein	Chugani et al. (2001); Ledgham et al. (2003b)
QslA	Negative regulator (anti-activator) of LasR and PqsR proteins	Seet and Zhang (2011)
QteE	Negative post-translational regulator of LasR and RhlR	Siehnel et al. (2010)
RpoN	Negative transcriptional regulator of *lasRI* and *rhlRI*	Heurlier et al. (2003); Thompson et al. (2003)
RpoS	Negative transcriptional regulator of *rhlI*	Latifi et al. (1996); Schuster et al. (2004); Whiteley et al. (2000)
RsaL	Negative transcriptional regulator of *lasI*	Bertani and Venturi (2004); de Kievit et al. (1999)
RsmA	Negative transcriptional regulator of *lasI*	Pessi et al. (2001)
Vfr	Positive transcriptional regulator of *lasR* and *rhlR*	Albus et al. (1997)
VpsR	Positive transcriptional regulator of *lasI*	Juhas et al. (2004)

### Quinolone-based intercellular signaling

The third QS signal, PQS, was purified and characterized in 1999 by Pesci and co-workers when they observed that spent culture media from wild type PAO1 causes a dramatic induction of *lasB* expression in a *lasR* mutant of *P. aeruginosa*, which could not be mimicked by OdDHL or BHL (Pesci et al., 1999). PQS is structurally identified as 2-heptyl-3-hydroxy-4-quinolone, and it is chemically unique from the AHL signals of the *las* and *rhl* systems. Originally studied as an antibacterial molecule (Cornforth and James, 1956; Lightbown and Jackson, 1956), this is the first instance that a 4-quinolone compound was reported as a signalling molecule in bacteria. The PQS synthesis cluster has been identified to consist of *pqsABCD*, *phnAB* and *pqsH* (Gallagher et al., 2002). Shortly after the identification of PQS signal, the receptor PqsR (then known as MvfR) has been implicated in the regulation of PQS production (Cao et al., 2001). PqsA is an anthranilate-coenzyme A ligase (Coleman et al., 2008; Gallagher et al., 2002), which activates anthranilate to form anthraniloyl-coenzyme A, initiating the first step of the PQS biosynthesis. A *pqsA* mutant does not produce any akyl-quinolones (AQs) (Deziel et al., 2004). PqsB, PqsC and PqsD are probable 3-oxoacyl-(acyl carrier protein) synthases and they mediate the conversion of anthranilate into 2-heptyl-4-quinolone (HHQ) by incorporation of β-ketodecanoic acid (Deziel et al., 2004; Gallagher et al., 2002). HHQ is the precursor of PQS and can be intercellularly transmitted between *P. aeruginosa* cells. HHQ is converted into PQS by the action of PqsH, a putative flavin-dependent monooxygenase that purportedly hydroxylates HHQ at the 3-position (Deziel et al., 2004; Dubern and Diggle, 2008; Gallagher et al., 2002; Schertzer et al., 2009). The transcription of *pqsH* is controlled by LasR, implying that the PQS system is controlled by the *las* system (Schertzer et al., 2009). PqsL is also predicted to be a monooxygenase and is most likely to be involved in the synthesis of the AQ *N-*oxides, (e.g. 4-hydroxy-2-heptylquinoline-*N-*oxide, HQNO) (Lépine et al., 2004). Disruption in PqsL caused an overproduction of PQS (D’Argenio et al., 2002), probably owing to a blocked AQ *N-*oxide pathway which leads to an accumulation of HHQ (Deziel et al., 2004; Lépine et al., 2004). In certain strains of *P. aeruginosa*, accumulation of PQS and HHQ leads to autolysis and cell death (D’Argenio et al., 2002; D’Argenio et al., 2007; Whitchurch et al., 2005). The role of PqsE remains largely unknown, which is a probable metallo-β-lactamase. Mutation of *pqsE* does not affect PQS biosynthesis (Gallagher et al., 2002), but the mutants failed to respond to PQS (Diggle et al., 2003; Farrow et al., 2008; Gallagher et al., 2002), and did not express the PQS-controlled phenotypes such as pyocyanin and PA-IL lectin production. In contrast, overexpression of PqsE alone led to enhanced pyocyanin and rhamnolipid production, which is otherwise dependent on the PQS signaling system (Farrow et al., 2008). These puzzling phenomena need to be further investigated for elucidating the role of PqsE in the bacterial physiology and virulence.

PqsR is a LysR-type transcriptional regulator that binds to the promoter region of *pqsABCDE* operon and directly controls the expression of the operon (Cao et al., 2001; Gallagher et al., 2002). The expression of *pqsR* is in turn controlled by LasR/OdDHL (Camilli and Bassler, 2006). PqsR is the cognate receptor of PQS and also its co-inducer, as the activity of PqsR in inducing the expression of *pqsABCDE* is dramatically increased when PQS is bound by the receptor (Wade et al., 2005; Xiao et al., 2006b). HHQ was also found to be able to bind to and induce the expression of PqsR, though it does so with ~ 100-fold less potency than PQS (Wade et al., 2005; Xiao et al., 2006a). Mutation of *pqsR* resulted in non-production of any AQs and pyocyanin (Cao et al., 2001; Gallagher et al., 2002; Schertzer et al., 2009; von Bodman et al., 2008), indicating that PqsR is essential for executing PQS signal transduction.

The importance of *pqs* signaling system in the bacterial infection has been illustrated by a range of studies. Null mutation of the *pqs* system resulted in reduced biofilm formation and decreased production of virulence factors such as pyocyanin, elastase, PA-IL lectin and rhamnolipids (Cao et al., 2001; Diggle et al., 2003; Rahme et al., 2000; Rahme et al., 1997). PQS is also required for full virulence towards plants (Cao et al., 2001), nematodes (Gallagher et al., 2002) and mice (Cao et al., 2001; Lau et al., 2004). In burn-wound mouse models, the killing abilities of *pqsA* are attenuated compared to the wild type parental strain (Déziel et al., 2005; Xiao et al., 2006b). Intriguingly, the *pqsH* mutant did not result in a decrease in virulence in burn-wound mouse model (Xiao et al., 2006b), but displayed a reduced killing on nematodes (Gallagher et al., 2002), hence the importance of PQS in regulation of virulence remains debatable. PQS, its precursor HHQ, and the derivative HQNO (4-hydroxy-2-heptylquinoline-N-oxide), are often found in the sputum, bronchoalveolar fluid and mucopurulent fluid of cystic fibrosis sufferers (Collier et al., 2002). Taken together, this could suggest that the precursors of PQS may play an equally important role as PQS in virulence and infections.

### An integrated QS system

**Figure 2:**
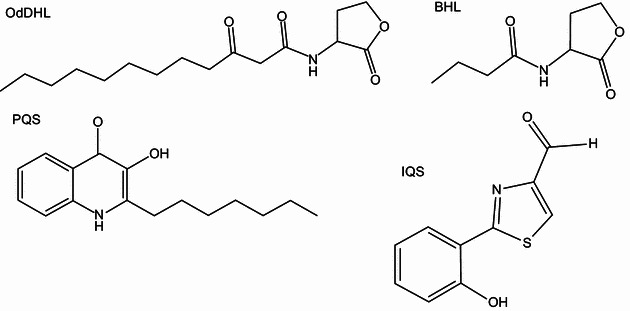
**Structures of***P. aeruginosa***quorum sensing (QS) signals**. Clockwise from left, *N*-(3-oxododecanoyl)-homoserine lactone (OdDHL); *N*-butyrylhomoserine lactone (BHL); 2-heptyl-3-hydroxy-4-quinolone (*Pseudomonas* Quinolone Signal, PQS); 2-(2-hydroxyphenyl)-thiazole-4-carbaldehyde (Integrated Quorum Sensing Signal, IQS)

Recently, a fourth inter-cellular communication signal has been discovered to be capable of integrating environmental stress cues with the quorum sensing network (Lee et al., 2013). Named as IQS, it belongs to a new class of quorum sensing signal molecules and was structurally established to be 2-(2-hydroxyphenyl)-thiazole-4-carbaldehyde. The genes that are involved in IQS synthesis are a non-ribosomal peptide synthase gene cluster *ambBCDE*. When disrupted, it caused a decrease in the production of PQS and BHL signals, as well as the virulence factors such as pyocyanin, rhamnolipids and elastase. Upon addition of 10 nmol/L IQS to the mutants, these phenotypes could be restored fully, indicating that IQS is a potent inter-cellular communication signal compared with its counterparts (Fig. 2). Further, IQS has been shown to contribute to the full virulence of *P. aeruginosa* in four different animal host models (mouse, zebrafish, fruitfly and nematode), highlighting the important roles of this new QS system in modulation of bacterial pathogenesis. Importantly, under phosphate depletion stress conditions, IQS was demonstrated to be able to partially take over the functions of the central *las* system (Lee et al., 2013), providing critical clues in understanding the puzzling phenomenon that the clinical isolates of *P. aeruginosa* frequently harbour mutated *lasI* or *lasR* genes (Ciofu et al., 2010; D’Argenio et al., 2007; Hoffman et al., 2009; Smith et al., 2006).

### Interconnection between the four QS systems

The QS circuits in *P. aeruginosa* are organized in a hierarchical manner. At the top of the signalling hierarchy is the *las* system. When activated by OdDHL, LasR-OdDHL complex multimerizes and activates the transcription of *rhlR*, *rhlI*, *lasI* (hence a positive feedback loop), and other virulence genes that are part of its regulon (Kiratisin et al., 2002; Latifi et al., 1996; Pesci et al., 1997). The RhlR-BHL complex also dimerizes and similarly activates the expression of its own regulon and *rhlI*, forming the second positive feedback loop (Ventre et al., 2003; Winson et al., 1995). LasR-OdDHL also positively regulates PqsR, the transcriptional regulator of the HHQ/PQS biosynthesis operon *pqsABCD*, as well as the expression of *pqsH*, the gene encoding the final converting enzyme of PQS from HHQ (Deziel et al., 2004; Gallagher et al., 2002; Xiao et al., 2006a). PQS, in turn, was found to be able to enhance the transcription of *rhlI*, thus influencing BHL production and the overall expression of the *rhl* QS system, thus indirectly modulating the *rhl*-dependent phenotypes (McKnight et al., 2000; Pesci et al., 1999). Interestingly, *pqsR* and *pqsABCDE* expression is inhibited by RhlR/BHL (Cao et al., 2001), suggesting that the ratio of the concentrations between OdDHL and BHL play a decisive role in the dominance of the *pqs* signaling system (Cao et al., 2001).

**Figure 3:**
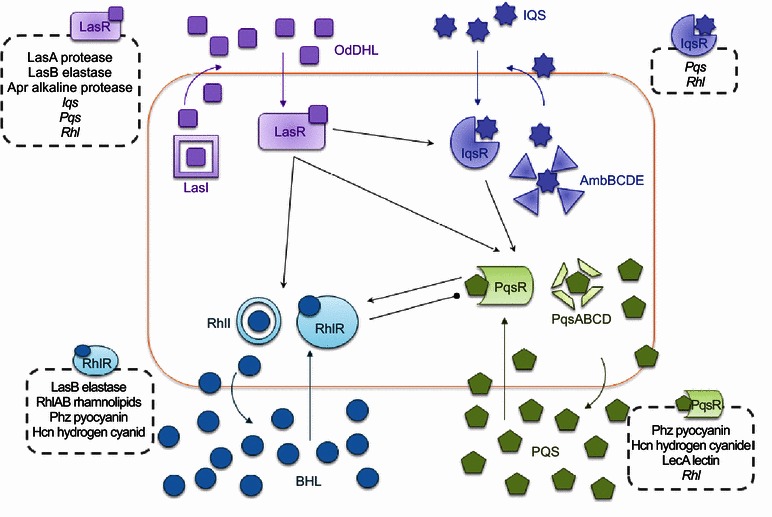
**Schematic representation of the four QS signaling networks in***P. aeruginosa***and their respective regulons**. Arrows indicate a stimulatory effect. Perpendicular lines indicate an inhibitory effect

With *las* governing the expression of both *pqs* and *rhl* systems, it was often described as being at the top of the QS hierarchy. The *rhl* system on the other hand, is under the control of both *las* and *pqs*, yet many QS-dependent virulence factors are predominantly activated by RhlR-BHL (Latifi et al., 1995; Schuster and Greenberg, 2007; Schuster et al., 2004; Whiteley et al., 1999; Winzer et al., 2000), thus the *rhl* system functions like a workhorse for the QS command. Since LasR-OdDHL controls the onset and activation of both the *pqs* and *rhl* QS circuits, these systems therefore represent a step-wise activation cascade that will be triggered by attainment of a “quorum” in *P. aeruginosa* cultures. The recently identified IQS was also found to be tightly controlled by LasRI under rich medium conditions. Disruption of either *lasR* or *lasI* completely abrogates the expression of *ambBCDE* and the production of IQS (Lee et al., 2013) (Fig. 3).

However, exceptions do occur. The *lasR* mutants were found to have a delayed production of PQS, instead of having an abolished PQS system as previously thought, and PQS could also overcome the dependency on LasR in activating the expression of *rhl* QS system and production of downstream virulence factors (Diggle et al., 2003). It was subsequently discovered that this could be due to the effects of RhlR, as the *lasR* and *rhlR* double mutant had barely any detectable PQS, but when *rhlR* was overexpressed, the production of PQS, as well as virulence factors such as LasB elastase and LasA protease, are restored (Dekimpe and Deziel, 2009). RhlR was also shown to upregulate the expression of *lasI*, the most-specific LasR-regulated gene, and OdDHL production was consequently increased (Dekimpe and Deziel, 2009). This indicates that compensation by the *rhl* QS system could override this hierarchy and maintain the expression of QS-dependent virulence factors in spite of a non-functional central *las* system. Similarly, the dominance of *las* on IQS signal production was reversed when *P. aeruginosa* was subjected to phosphate depletion stress, and the *iqs* system could up-regulate the expression of *pqs* and *rhl* systems and the production of QS-dependent virulence factors in the *lasI* or *lasR* mutant (Lee et al., 2013). Low phosphate levels also elevate IQS production in wild type *P. aeruginosa* (Lee et al., 2013). These findings highlight the importance of environmental factors in modulating the bacterial QS systems and the plasticity of the QS networks in accommodation and exploitation of environmental changes for the benefit of bacterial pathogens. The next section is dedicated to discussion of such examples in details with the aim to shed light on understanding the complicated and sophisticated QS regulatory mechanisms in *P. aeruginosa*.

## Environmental triggers and the QS responses

Evidence is accumulating that environmental stress conditions could exert substantial influence on the QS systems of *P. aeruginosa*. Starvation, phosphate and iron depletion are known to promote the expression and activity of RhlR in the absence of *lasR* (Jensen et al., 2006; Van Delden et al., 1998). More recently, it was found that phosphate depletion could induce IQS production even in the absence of functional *las* system (Lee et al., 2013). This discovery is clinically significant as substantial amount of *P. aeruginosa* chronic infection isolates bear a loss-of-function *las* system (Cabrol et al., 2003; Denervaud et al., 2004; Hamood et al., 1996; Schaber et al., 2004; Smith et al., 2006). The roles and the molecular mechanisms with which various environmental cues and host immune factors modulate the QS systems of *P. aeruginosa* will be discussed separately in the following sections.

### Phosphate-depletion stress

Phosphate is essential for all living cells owing to its key roles in signal transduction reactions such as phospho-relay, and as an essential component of the energy molecule ATP, nucleotides, phospholipids and other important biomolecules. Foreseeably, bacterial pathogens may encounter strong competition for free phosphates from host cells during the process of pathogen-host interaction. Therefore, the ability to withstand phosphate starvation and the response mechanisms of harnessing phosphate from external sources is critical for *P. aeruginosa* survival and establishment of infections. As a result, phosphate-depletion stress has been shown to have far-reaching effects on QS signalling profiles, gene expression, physiology and virulence of bacterial pathogens (Chugani and Greenberg, 2007; Frisk et al., 2004; Jensen et al., 2006; Lee et al., 2013; Zaborin et al., 2009).

When facing with phosphate limitation, *P. aeruginosa* exhibits increased swarming motility and cytotoxicity towards the human bronchial epithelial cell line 16HBE14o- (Bains et al., 2012), attesting to the strong responses phosphate deprivation could elicit from the pathogen. Additionally, phosphate depletion stress was shown to prompt the up-regulation of iron chelator pyoverdine biosynthesis, which in turn, could result in the inactivation of the phosphate acquisition pathway. When the pyoverdine signalling pathway was interrupted, pyochelin biosynthesis was in turn increased as compensation (Zaborin et al., 2009). This resulted in high amounts of ferric ions to be acquired. Coupled with the dramatic increase in PQS production (part of the phosphate starvation response), the lethal PQS-Fe(III) red coloured complex was formed. When ingested, the red-spotted *P. aeruginosa* caused rapid mortality in *C. elegans*, a phenomenon known as “red death” (Zaborin et al., 2009). Such signalling cross-talk demonstrates the interconnectivity between the phosphate and iron acquisition systems in *P. aeruginosa*, the investment in resources the bacteria makes to maintain their homeostasis, and the deleterious effects on the host when the fine balance is tipped.

The lack of phosphate also dramatically activates the expression of *pqsR* and the PqsR-regulated *pqsABCDE* and *phnAB* genes. Along with the enhanced *pqs* system, the expression of QS-associated virulence genes responsible for the synthesis of rhamnolipids, phenazines, cyanide, exotoxin A and LasA protease are similarly induced (Bains et al., 2012; Zaborin et al., 2009). This was thought to lead to the acute mortality rate of the host organism *Caenorhabditis elegans* after being infected by *P. aeruginosa* that were grown in phosphate starvation medium (Zaborin et al., 2009). These observations correlate and could well be explained by our current knowledge on IQS. With depletion in phosphate, expression of *iqs* system is induced (Lee et al., 2013), which in turn triggers an up-regulation of the downstream *pqs* and *rhl* QS systems, and eventually, an observed boost in QS-associated virulence factors production and killing rates.

It is crucial to note that the two-component sensor-response regulator system PhoBR plays an indispensable role in detection and signal transduction of phosphate stress cues (Anba et al., 1990; Filloux et al., 1988; Hsieh and Wanner, 2010), as disruption of *phoB* completely abolished the virulence of *P. aeruginosa* towards *C. elegans* (Zaborin et al., 2009), and dramatically diminished its swarming motility and cytotoxicity (Bains et al., 2012). PhoB (and the *pho* regulon) was also shown to participate in the inhibition of biofilm formation, c-di-GMP signal degradation and repression of the type III secretion systems (Haddad et al., 2009), all of which could significantly affect the clinical outcome during *P. aeruginosa* infections (Abe et al., 2005; Costerton, 2001; Hauser et al., 2002; Hueck, 1998; Roy-Burman et al., 2001). The *phoB* mutant grows poorly in low phosphate medium and failed to produce the QS-dependent virulence factor pyocyanin (Lee and Zhang, unpublished data). Remarkably, PhoBR is indispensable for coordinating the *las*-independent, phosphate-dependent IQS signalling activation, wherein the “IQS phenotype” would be abolished in a *phoB* mutant (Lee et al., 2013). The PhoBR-IQS loop could also explain the observations by Jensen and co-workers, who reported that low phosphate prompted an enhancement of the *rhl* QS system even when *las* was functionally absent and this is coordinated by PhoB (Jensen et al., 2006).

### Iron and PQS signaling system

Unlike phosphate, the modulatory effect of iron starvation on *P. aeruginosa* QS networks appears to be less direct. A deficiency in iron does lead to notable increases in the expression of genes involved in iron acquisition (ferric uptake siderophores, pyochelin and pyoverdine; ferrous iron transporters like haem and *feo*), exoenzymes that could cleave iron-bound host proteins (alkaline protease, *lasB* elastase) and other redox enzymes and toxins (exotoxin A) (Ochsner et al., 2002). Further, the iron depletion stress response was found to lead to an inhibition of oxygen transfer from the atmosphere to liquid *P. aeruginosa* cultures, thus protecting bacteria cells from oxidative stress. Production of the virulence factor LasB elastase is also significantly increased in these iron depletion cultures (Kim et al., 2003). Although some of the upregulated virulence factors, like alkaline protease and elastase, are known to be regulated by the QS systems of *P. aeruginosa* (see Table 1), a direct link between iron deprivation and up-regulation of central QS genes such as *lasI*, *lasR*, *rhlI* or *rhlR* has yet to be found. In a report by Diggle and co-workers, the PQS molecules were found to function as an iron trap when secreted into the extracellular milieu of *P. aeruginosa* (Diggle et al., 2007). This was hypothesized to serve the purpose of storing up free ferric ions which could subsequently be internalized into the cells by the siderophores, in order to safeguard against a sudden dip in iron concentration. Iron starvation could also trigger a Fur-dependent de-repression of the small regulatory RNAs *prrF1* and *prrF2* expression. PrrF1 and PrrF2 bind to and inhibit the expression of *antABC* genes which encode for the anthranilate degradation enzymes AntABC. Since anthranilate is the precursor of PQS biosynthesis, inhibition of its degradation could lead to accumulation of anthranilate, which consequently elevates the concentration of HHQ and PQS in the bacteria cells. This in turn might boost the PQS-PqsR signaling pathway. PqsR was also found to inhibit *antABC* expression, albeit in a PrrF1,2-independent manner (Oglesby et al., 2008). Taken together, the above findings seem to suggest that iron depletion stress may modulate bacterial virulence through the *pqs* system, which awaits further investigations.

### ANR and oxygen deprivation

Low oxygen tension is a key factor affecting cyanide biosynthesis (cyanogenesis) in *P. aeruginosa* (Castric, 1994; Castric, 1983). The final product, hydrogen cyanide (HCN), is a highly potent extracellular virulence factor and contributes to high mortality rates during infection of host organisms (Ryall et al., 2008; Solomonson, 1981). Additionally, increase in *P. aeruginosa* cell density was also shown to remarkably elevate expression of *hcnABC*, the synthase genes for HCN, and reaches its optimum levels during the transit from exponential to stationary growth phase of the bacteria (Castric et al., 1979). This may suggest a cooperative link between oxygen deprivation and QS in the regulatory mechanism of cyanogenesis, which was subsequently demonstrated through characterization of ANR, a transcriptional regulator associated with bacterial anaerobic growth.

ANR, which is converted into its active form when oxygen tension is low, is a key regulator controlling the expression of arginine deiminase and nitrate reductase. ANR belongs to the FNR (fumarate and nitrate reductase regulator) family of transcriptional regulators and is the main transcriptional regulator that acts in parallel with the QS systems for the expression of hydrogen cyanide biosynthesis genes (Pessi and Haas, 2000). ANR, together with LasR-OdDHL or RhlR-BHL, bind to the promoter region of the *hcnABC* cluster, exhibiting a synergistic effect brought upon by oxygen limitation stress. Further, the PRODORIC promoter analysis programme predicted the FNR/ANR binding consensus sequences in up to 25% of the predicted QS-controlled promoters, implying that ANR might be an important co-regulator of the QS-dependent virulence genes in anaerobic environments (Schuster and Greenberg, 2006).

### Starvation stress

When exposed to unfavourable environments and nutrient starvation, *P. aeruginosa* must rapidly cope and elicit a prompt response to modify their metabolic profiles for survival. This process is termed as the stringent response and brings about diverse effects ranging from inhibition of growth processes to cell division arrest (Joseleau-Petit et al., 1999; Svitil et al., 1993) and more importantly, a premature activation of the *P. aeruginosa* QS systems that is independent of cell-density (van Delden et al., 2001). The QS signals BHL and *N-*hexanoyl-homoserine lactone (HHL) are prematurely produced and PQS synthesis inhibited (Baysse et al., 2005). The spike in BHL QS signal is likely to result in the concomitant increase in production of downstream virulence factors elastase and rhamnolipids (Schafhauser et al., 2014).

The QS-based response is mediated by the stringent response protein RelA. In face of amino acid shortage, uncharged tRNA triggers the activity of the ribosome-associated RelA, which in turn synthesizes ppGpp (nucleotide guanosine 3’,5’-bisdiphosphate), an intracellular signal that enables the bacteria cell to self-perceive their inability in synthesis of proteins (Gentry and Cashel, 1996). When overexpressed, RelA leads to early transcriptional expression of the *lasR* and *rhlR* genes, as well as production of QS signals OdDHL and BHL (van Delden et al., 2001), hence leading to the overproduction of the aforementioned QS-dependent virulence factors. Furthermore, RelA and ppGpp was also shown to coordinate the stress response associated with alterations in membrane phospholipid composition and loss of membrane fluidity. When the phospholipid biosynthesis protein LptA was deleted, an increase in *relA* expression and ppGpp production was observed, which resulted in a premature activation of BHL and HHL QS signals biosynthesis (Baysse et al., 2005).

In a recent study, Schafhauser and co-workers observed that the synthesis of the starvation signal ppGpp negatively regulates the biosynthesis of HHQ and PQS signals, and is required for full expression of both the *las* and *rhl* QS systems (Schafhauser et al., 2014). In the *relA* and *spoT* double mutant that is unable to synthesize ppGpp, both the *las* and *rhl* QS systems are down-regulated, and the production of QS-dependent virulence factors rhamnolipid and elastase are reduced (Schafhauser et al., 2014). Whilst it has been previously reported that ppGpp increases the expression of LasR and RhlR and the resultant downstream factors (Baysse et al., 2005; van Delden et al., 2001), repression on the *pqs* system by ppGpp is somewhat unexpected. More experiments are required to investigate on the significance of this selective dampening of the *pqs* system.

### Response to host factors

It has been traditionally thought that opportunistic pathogens such as *Pseudomonas aeruginosa* invade hosts with a weakened immune system or attenuated epithelial barrier in a passive manner, until an important observation was made by Wu and colleagues that *P. aeruginosa* major outer-membrane protein OprF is able to recognize and bind to human T cell-based cytokine interferon gamma (IFN-γ). This in turn activates the *rhl* QS system and substantially enhances the expression of *lecA* and production of its encoded virulence protein, galactophilic lectin. Pyocyanin, an additional QS-regulated virulence factor, was also found to be up-regulated in the presence of IFN-γ (Wu et al., 2005). Although IFN-γ was the only cytokine found to activate the *rhl* QS system and it is not known whether and if yes, how the upstream *las* and *pqs* networks are affected, this work presents a direct evidence of the interactions between host-derived immune factors and bacterial membrane proteins, which consequently leads to QS-based responses. In another example, dynorphin, an endogenous κ-receptor agonist, was found to penetrate the bacterial membrane and potently induce the expression of *pqsR* and pqsABCDE, and lead to increased biosynthesis of PQS, HHQ and the related derivative HQNO. The growth advantage against probiotic gut microorganisms *Lactobacillus* spp. and virulence towards *C. elegans* is also remarkably enhanced when *P. aeruginosa* is exposed to dynorphin (Zaborina et al., 2007). This finding is of particular significance to *P. aeruginosa* caused gut infections as dynorphin is usually in high concentrations in the intestinal mucosa and epithelial cells, attesting to the remarkable mechanisms utilized by the bacteria to enhance virulence by integrating host opioids into its existing QS circuitry.

Further, human hormones, particularly the C-type natriuretic peptide (CNP) that is produced by endothelial cells and used for maintaining body fluid homeostasis and blood pressure control, was demonstrated to have positive effects on *P. aeruginosa* virulence. Through activation of the *P. aeruginosa* membrane natriuretic peptides sensor, CNP induces a rise in intracellular cAMP concentration and lead to the activation of the global virulence activator Vfr, which either alone or together with another regulator PtxR, enhances the synthesis of QS signals OdDHL and BHL, and inhibits the production of PQS. Vfr also drives the increased expression of virulence factors hydrogen cyanide and lipopolysaccharide, thereby elevating the mortality rate in *C. elegans* infected with CNP-treated *P. aeruginosa* (Blier et al., 2011).

Most recently, the human host defence peptide LL-37, the only cathelicidin class of cationic antimicrobial peptides synthesized by phagocytes, epithelial cells and keratinocytes, was revealed to exert a positive effect on *P. aeruginosa* QS and virulence profiles. When stimulated by exogenous LL-37 at physiological concentrations, *P. aeruginosa* exhibits heightened production of virulence factors pyocyanin, hydrogen cyanide, elastase and rhamnolipids. The PQS signal level is also elevated. LL-37 was also found to decrease the susceptibility of the bacteria to gentamicin and ciprofloxacin antibiotics. These phenotypes were suggested to be mediated by the quinolone response protein and virulence regulator PqsE (Strempel et al., 2013).

## Summary and perspectives

**Figure 4:**
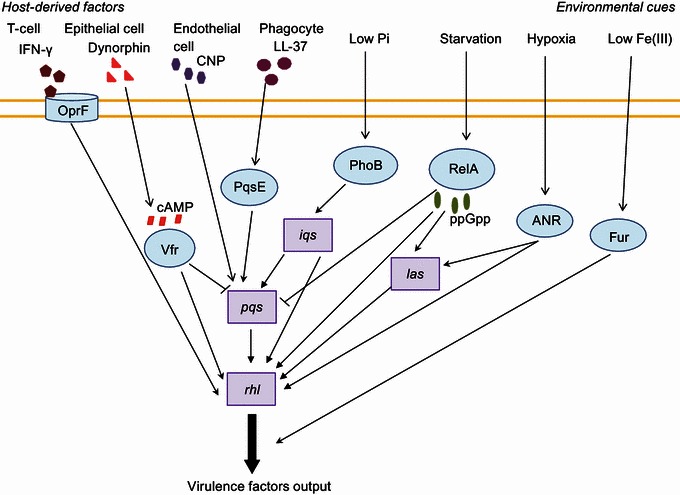
**Schematic representation of how environmental conditions and host factors influence the***P. aeruginosa***QS signaling hierarchy**. For simplicity, the QS systems are represented as a whole unit, namely, *las*, *iqs*, *pqs* and *rhl*

*Pseudomonas aeruginosa* is one of the most notorious opportunistic human pathogens as it employs a variety of virulence factors and mechanisms during infection (Fig. 1). The type of virulence pathways activated is often dependent on the environment conditions and stresses the bacteria encounter. Extensive research over the past two decades has documented numerous instances of environmental cues including the biostresses of host origin, which could dramatically influence the virulence phenotypes of *P. aeruginosa*. The findings from recent research progresses suggest that these effects could largely be through modulation of the bacterial QS network, which comprises at least four QS signaling mechanisms including *las*, *iqs*, *pqs* and *rhl*. In particular, the most recently identified IQS highlights how a bacterial QS system could integrate environmental cues with bacterial quorum information. These four systems interact closely with one another giving rise to an intricately linked intercellular communication network. Such a complicated and multi-component QS network may enable *P. aeruginosa* to accommodate various environmental cues and biostresses (Fig. 4).

Previous efforts in the design of anti-QS therapeutics were focused primarily on inhibition of the *las* system (Borlee et al., 2010; Mattmann and Blackwell, 2010). However, in light of the recent discovery that IQS could replace the functions of *las* in conditions that closely mimics host infection (Lee et al., 2013), coupled with the high mutation frequencies of *lasR* typical of *P. aeruginosa* clinical isolates (Ciofu et al., 2010; D’Argenio et al., 2007; Hoffman et al., 2009; Smith et al., 2006), it becomes clear that the ongoing strategies targeting the *las* system is insufficient, and that the prevalence of IQS system in clinical isolates should be evaluated to ensure development of potent anti-QS therapeutics. Furthermore, we should also keep in mind that there are many unknowns that require further investigations for clear understanding of how the bacterial QS network could act on various environmental cues in regulation of bacterial virulence and biofilm formation. For example, it is not clear how IQS could regulate the downstream *pqs* and *rhl* signaling systems and what is the impact of *iqs* system on the virulence of clinical isolates. Similarly, much remains to be done in understanding whether and if yes, how environmental cues could modulate the *las*, *pqs* and *rhl* systems. Recognition of how the external stressors change the way the QS network is connected may generate tremendous impact on the perspective from which therapeutic interventions could be developed, especially those environmental cues almost always encountered by *P. aeruginosa* during infections of the host. For instance, successful establishment of an infection and colonization of the cystic fibrosis lung chambers would require *P. aeruginosa* strains to sense, withstand and respond to deprivation of iron, phosphate, and attacks by lung macrophage-derived factors (Campodonico et al., 2008; Konings et al., 2013; Krieg et al., 1988). Then, as the pathogen transits into a long-term, chronic infection mode, the stresses of living within a biofilm matrix may include oxygen deprivation and nutrient limitation (Jackson et al., 2013; Sauer et al., 2004). Investigation along this line will further advance our understanding of the complicated and sophisticated QS regulatory mechanisms and may continue to generate unexpected interesting findings.

## Abbreviations

AHL: acylhomoserine lactone
CNP: C-type natriuretic peptide
HCN: hydrogen cyanide
IFN-γ: interferon gamma
IQS: integrated quorum sensing system
QS: quorum sensing
QSIs: quorum sensing inhibitors
